# Clinical significance of eukaryotic translation initiation factor 5A2 in papillary thyroid cancer

**DOI:** 10.1080/21655979.2020.1848753

**Published:** 2020-11-29

**Authors:** Hongmei Zhang, Kejun Zhang, Liang Ning, Dong Chen, Fengyun Hao, Peng Li

**Affiliations:** a Department of Surgical Day Ward, The Affiliated Hospital of Qingdao University, Qingdao, Shandong, China; b Department of Thyroid Surgery, The Affiliated Hospital of Qingdao University, Qingdao, Shandong, China; c Department of Pathology, The Affiliated Hospital of Qingdao University, Qingdao, Shandong, China; d Department of Endocrinology, The Affiliated Hospital of Qingdao University, Qingdao, Shandong, China

**Keywords:** Papillary thyroid cancer, metastasis, apoptosis, prognostic marker, eukaryotic translation initiation factor 5A2

## Abstract

Eukaryotic translation initiation factor 5A2 (eIF5A2) plays an important role in tumor progression and prognosis evaluation. However, the potential role of eIF5A2 in human papillary thyroid cancer (PTC) is unknown. In this study, we aim to investigate the association between eIF5A2 expression and PTC clinical outcomes and underlying its Biological function in PTC cells *in vitro* and *in vivo*. The expression of eIF5A2 was examined by immunohistochemistry in PTC tissues and its adjacent tissue (n = 39) from 207 PTC patients. Functional analysis of eIF5A2 was performed in PTC cell lines *in vitro* and *in vivo*. The results showed that eIF5A2 was overexpressed in PTC tissues compared with the adjacent tissues. Enhanced eIF5A2 expression was significantly correlated with extrathyroidal extension (*p* = 0.012), lymph node metastasis (*p* = 0.002), TNM stage (*p* = 0.006), T classification (p = 0.047) and BRAF V600E mutation (*p* = 0.036). EIF5A2 inhibition prevented PTC cell growth, invasiveness and migration and induced cell apoptosis *in vitro*. Furthermore, eIF5A2 depletion inhibited tumor growth and metastasis *in vivo*. The data indicated that eIF5A2 could be employed as a novel prognostic marker and effective therapeutic target for PTC.

## Graphical abstract

**Figure:**
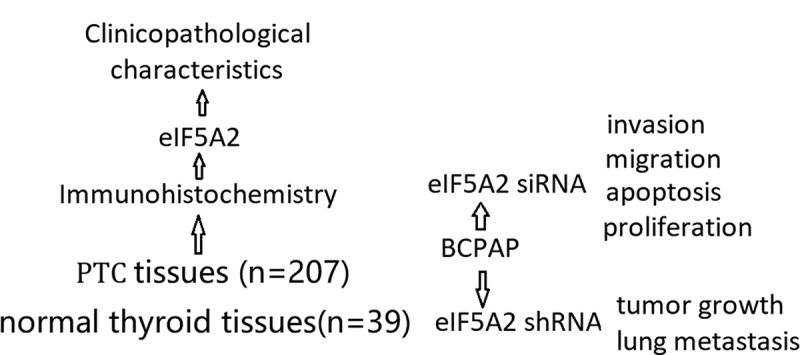

## Introduction

Thyroid cancer is a common endocrine malignancy, of which papillary thyroid carcinoma (PTC) accounts for 85–90%. However, no reliable and specific markers are used for the diagnosis of PTC [1]. Ultrasound (US) diagnosis is the most commonly used clinical method to diagnose thyroid cancer and central and lateral neck lymph nodes metastasis, but the sensitivity and specificity in detecting abnormal lymph nodes is low and current US has an accuracy of only 72.6% [2]. Ultrasound-guided fine needle aspiration (FNA) is the most commonly used method currently to evaluate and triage patients with thyroid nodules and lymph nodes. However, 20% and 30% of FNA biopsy samples can not acquire accurate diagnosis.

For more than twenty years, identifying suitable molecular markers to guide surgery or to wait for patients with FNA biopsy (FNAB) with uncertain thyroid nodules has been the key to thyroid nodules research. Many potential genetic diagnostic markers, such as mutations in RAS, PIK3CA, PTEN, P53, TERT and BRAF genes and marker combinations have been studied in thyroid or lympy nodes and FNAB specimens [3–10]. However, no combination can significantly improve the clinical diagnosis rate of thyroid nodules. Molecular targeted therapies are also booming, such as BRAF inhibitor Vemurafenib and the MEK inhibitor selumetinib [11,12]. However, the toxic effects of the kinase inhibitors are inevitable. Therefore, the purpose of predictive biomarkers is to better select patients suitable for this type of targeted therapy and to evaluate the tumor’s response to drugs early.

Only two proteins in eukaryotic cells have the unique amino acid tyrosine, namely eIF5A1 and eIF5A2, which are covalently linked to the lysine 50 residues of these proteins through post-translational modifications on hypnosis, guided by two highly conserved and essential enzymes-deoxyhysupsine synthase (DHPS) and deoxyhysupsine hydroxylase (DOHH) [13]. Basic research and clinical evidence show that eIF5A2 gene amplification has been shown in many cancer tissues and cell lines [14]. Enhanced eIF5A2 expression correlates with poor prognosis and poor response for chemotherapeutic drugs in patients with cancers [15], suggesting that eIF5A is a useful biomarker in the prediction of cancer prognosis and drug response. Enhanced eIF5A2 has reported to initiate tumor formation, promote tumor cell metastasis and promote drug response, suggesting that targeting eIF5A2 could inhibit tumorigenesis and metastasis as well as enhances the sensitivity of drugs [16,17]. However, the clinical significance of eIF5A2 in patients with PTC and its biological function in PTC cells is unknown.

Here we investigate the expression of eIF5A2 in patients with PTC and the function in PTC cells *in vitro* and *in vivo*. We also determine whether eIF5A2 expression relates with clinical catchratics in patients with PTC. Finally, we determine its role in regulating the malignant cell phenotype in PTC cell lines.

## Materials and methods

### Cell line and culture

The PTC cell line BCPAP was purchased from the European Collection of Cell Cultures (ECACC, Salisbury, United Kingdom). It was maintained in 10% fetal bovine serum and 5% CO_2_ at 37°C.

### Patients

Primary papillary thyroid cancer (PTC) from 207 consecutive patients who underwent surgical therapy and adjacent tissue in 39 patients between 2018 and 2019 at the affiliated hospital of Qingdao University. Written informed consent was obtained from all patients. The Institutional Review Board of the affiliated hospital of Qingdao University approved the study. Detailed history was recorded and examination was done. No patients received neoadjuvant chemotherapy or radiotherapy. The age of the patients ranged from 17 to 76 years (mean age was 48.7 years). All these specimens were pathologically confirmed as PTC.

### Immunohistochemistry (IHC)

Formalin-fixed paraffin-embedded tissue sections from excised specimens were processed according to standard procedures. Immunohistochemical staining and positive expression judgment for eIF5A2 was performed as described previously [18,19].

### Western blot

A total of 20 µg protein was separated by 10% SDS-PAGE and transferred to a 0.2 µm polyvinylidene fluoride membrane using the Bio-Rad SemiDry instrument (Bio-Rad), and incubated with anti-eIF5A2 and anti-GAPDH (Santa Cruz, Shanghai, China) at 4°C overnight. After washing with 0.1% TBST for 15 minutes and performing 3 times, the membrane is coupled to the secondary stage of HRP Dilute the antibody at a ratio of 1:10,000 for 1 h at room temperature. Then, it was washed again with 0.1% TBST 3 times, 15 minutes each time. The enhanced chemiluminescence detection (ECL; GE, Healthcare, Piscataway, NJ) was used for semi-quantitative analysis.

### Lentiviral-mediated silencing of eIF5A2 gene in BCPAP cells

The PLVX-IRES-ZsGreen vector containing eIF5A2-short hairpin RNA (eIF5A2-shRNA) was designed and amplified using the standard method. The recombinant viruses were infected into BCPAP cells and Puromycin (0.4 μg/mL) was used to select stable transfected colonies.

### siRNA transfection

siRNA oligonucleotides were purchased from GenePharma (Shanghai, China). siRNA sequences against eIF5A2 were as follows: 5ʹ CAUUCAAGAUGGUUACCUUtt 3ʹ and 5ʹAAGGUAACCAUCUUGAAUGca 3ʹ. The siRNA oligonucleotides were transfected into BCPAP cells using lipofectamine™ 2000 (Invitrogen, Carlsbald, CA) as the manufacture’s instructure.

### Invasion assay

Cell invasion/migration was performed using BD BioCoat Matrigel Invasion Chambers (BD Biosciences) according to a previously described method [5]. Experiments were performed three times in triplicate.

### Cell viability assay

Cell viability was determined by the 3-(4, 5-dimethylthiazol-2-yl)-2,5-diphenyltetrazolium bromide (MTT) assay, using an assay kit as the manufacturer’s instructure. After 96 h incubation, cell viability was evaluated by adding 50 μl of 0.15% MTT (Sigma, St. Louis, MO) to each well.

### Clonogenic survival assay

Cells were plated at 500 cells/well into 6-well plates (Corning Inc., Corning, NY) and cultured for 10 days, then stained with crystal violet using the standard method. The colony formation rate was calculated as the (number of clones)/(number of seeded cells)×100.

### *Apoptosis assay* in vitro

Cell apoptosis was performed using an Annexin V-PE/7-AAD Apoptosis Detection Kit (KeyGEN BioTECH, Nanjing, China) according to the manufacturer’s instructions.

### *Tumor grow* in vivo

Lv-**eIF5A2**-shRNA cells (7 x 10^6^/0.2ul PBS) or Lv-NV cells (7 x 10^6^/0.2ul PBS) were injected sc into the flank of mice (n = 6). Tumor sizes were measured and calculated weekly for 5 weeks using a caliper. Tumors were retrieved and placed in 10% formalin for histology and TUNEL assay.

### *Lung metastasis* in vivo

4-6-week-old SCID mice (Shanghai Institute of Laboratory Animals, Chinese Academy of Sciences) were injected with 2 × 10^5^ BCPAP-Lv-**eIF5A2**-shRNA cells via the tail vein. The mice were killed under anesthesia after 4 weeks. The lungs were fixed in 4% polyoxymethylene.

### Histology and immunohistochemistry

Paraffin-embedding retrieved tumors were washed and dehydrated. 4 μm thick sections were mounted on poly-lysine slides, and stained with H&E for the light microscopy analysis. 2 μm thick sections were incubated with the anti-**eIF5A2 and** anti-Ki-67 antibody for immunohistochemistry staining as the manufacture’s protocol.

### TUNEL staining

Cell apoptosis was detected by Terminal Deoxynucleotidyltransferase (TdT)-Mediated dUTP Nick End Labeling (TUNEL) Staining (Roche Diagnostics, Guangzhou, China) using an in situ cell death detection kit, all according to the supplier’s instructions.

### Statistical analysis

Values were reported as means ± SD and compared by Student’s t-test or chi-squared test. Statistical analysis was performed using SPSS.22.0 (IBM SPSS, Armonk, NY, USA). P < 0.05 was considered as statistical significance. Each assay was repeated three independent times in triplicates.

## Results

### eIF5A2 is overexpressed in PTC tissues

**Figure 1.**
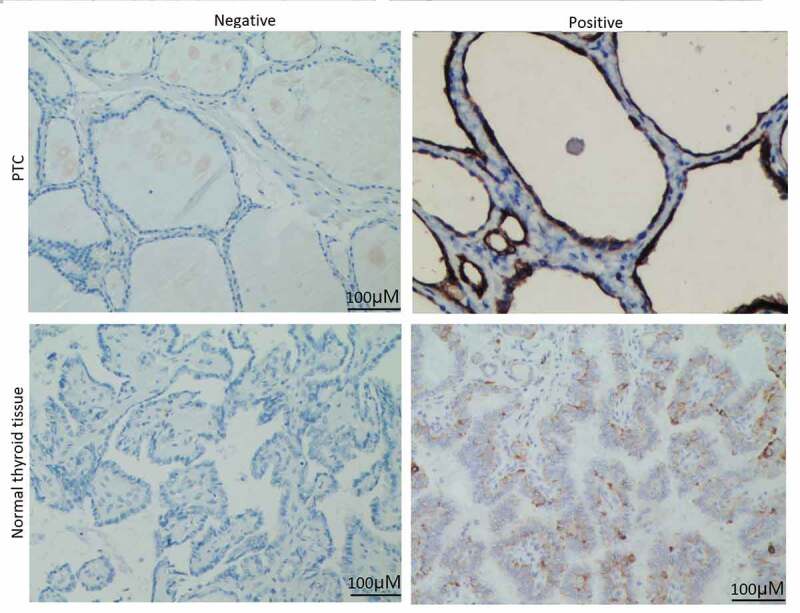
eIF5A2 is upregulated in human PTC samples. Representative images of eIF5A2 immunohistochemical staining in PTC and in normal thyroid tissue

We first analyzed the expression of eIF5A2 in 207 cases of PTC and 39 cases of adjacent tissues using immunohistochemical staining. As shown in Figure 1, positive expression of eIF5A2 was detected in 47.3% (98/207) of PTC tissues and 15.4% (6/39) of the adjacent tissues. eIF5A2 was overexpressed in PTC tissues compared to the adjacent tissues (*P* = 0.018).

### eIF5A2 expression and clinicopathological features in PTC

Table 1 summarizes the positive correlates of eIF5A2 expression with clinicopathological features in patients with PTC. eIF5A2 overexpression related with extrathyroidal extension (*p* = 0.012), lymph node metastasis (*p* = 0.002), TNM stage (*p* = 0.006), T classification (p = 0.047) and BRAF V600E mutation (*p* = 0.036). No significant differences were found between the two groups in terms of other clinicopathological variables, including patient age, gender, distant metastasis, Follicular variant and multifocality.

**Table 1.** Relationship between eIF5A2 and clinicopathological factors in PTC patients

Groups		eIF5A2 expression		
Gender	Number (*n* = 207)	Negative (*n* = 109)	Positive (*n* = 98)	*p*-Value
Male	57	28 (25.7%)	29 (29.6%)	NS
Female	150	81 (74.3%)	69 (70.4%)	
Age (Year)				
≤50	81	41 (37.6%)	40 (41%)	NS
>50	126	68 (62.4%)	58 (59%)	
Tumor size				
≤1 cm	97	49 (45.0%)	48 (49.0%)	NS
>1 cm	110	60 (55.0%)	50 (51.0%)	
Extrathyroidal extension				
Yes	78	32 (29.4%)	46 (46.9%)	0.012
No	129	77 (70.6%)	52 (53.1%)	
Distant metastasis				
Yes	6	1 (1%)	5 (5.1%)	0.29
No	201	108 (99%)	93 (94.9%)	
Lymph node metastasis				
Yes	90	32 (29.4%)	58 (59.2%)	0.002
No	117	77 (70.6%)	40 (40.8%)	
TNM stage				
I/II	173	101 (92.7%)	72 (73.5%)	0.006
III/IV	34	8 (7.3%)	26 (26.5%)	
T stage				
1–2	166	90 (82.6%)	76 (77.6%)	0.047
3–4	41	19 (17.4%)	22 (22.4%)	
BRAF V600E mutation				
Yes	89	33 (30%)	56 (57%)	0.036
No	118	86 (70%)	42 (43%)	
Multifocality				
Yes	68	34 (31.2%)	34 (34.7%)	NS
No	139	75 (68.8%)	64 (65.3%)	
Follicular variant				NS
Yes				
No				

### *Targeting eIF5A2 inhibits cell viability and induces cell apoptosis* in vitro

**Figure 2.**
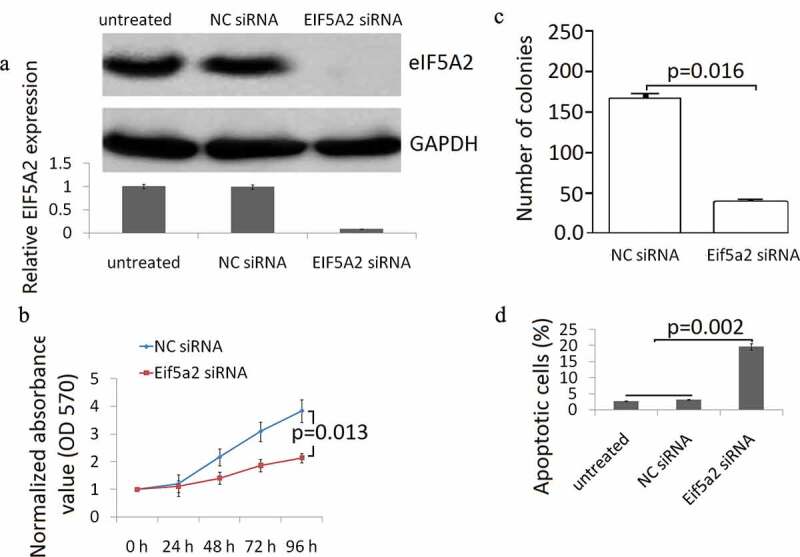
Knockdown of eIF5A2 suppresses BCPAP cell grow and induces apoptosis *in vitro*. (a) BCPAP cells were transiently transfected with either 20 nM siRNA (NC siRNA) control or eIF5A2 siRNA for 48 h. The cells were subjected to western blot assay for eIF5A2; (b) BCPAP cells were transiently transfected with either 20 nM siRNA (NC siRNA) control or eIF5A2 siRNA for 0–96 h. Cell viability was detected by MTT assay. Data represent at least three experiments performed in triplicate. (c) BCPAP cells were treated with either 20 nM siRNA (NC siRNA) control or eIF5A2 siRNA for 10 days and examined by colony formation assay. (d) BCPAP cells were treated with either 20 nM NC siRNA or eIF5A2 siRNA for 72 h, cell apoptosis was detected by flow cytometry

The knockdown of eIF5A2 expression in BCPAP cells was confirmed by Western blot analysis. High basal eIF5A2 expression was shown in BCPAP cells, eIF5A2 siRNA completely inhibited eIF5A2 expression in BCPAP cells, and no affect was shown in control NC siRNA transfected BCPAP cells (Figure 2(a)). MTT assay revealed that the eIF5A2 silencing resulted in a significant inhibition of cell viability (Figure 2(b)). The colony formation assay confirmed that eIF5A2 silencing reduced the number of colonies (Figure 2(c)). Flow cytometry results are presented in Figure 2(d). eIF5A2 silencing induced significant cell apoptosis in BCPAP cells compated with the BCPAP cells transfected with the control NCsiRNA.

### *Targeting eIF5A2 inhibits cell invasion and migration* in vitro

**Figure 3.**
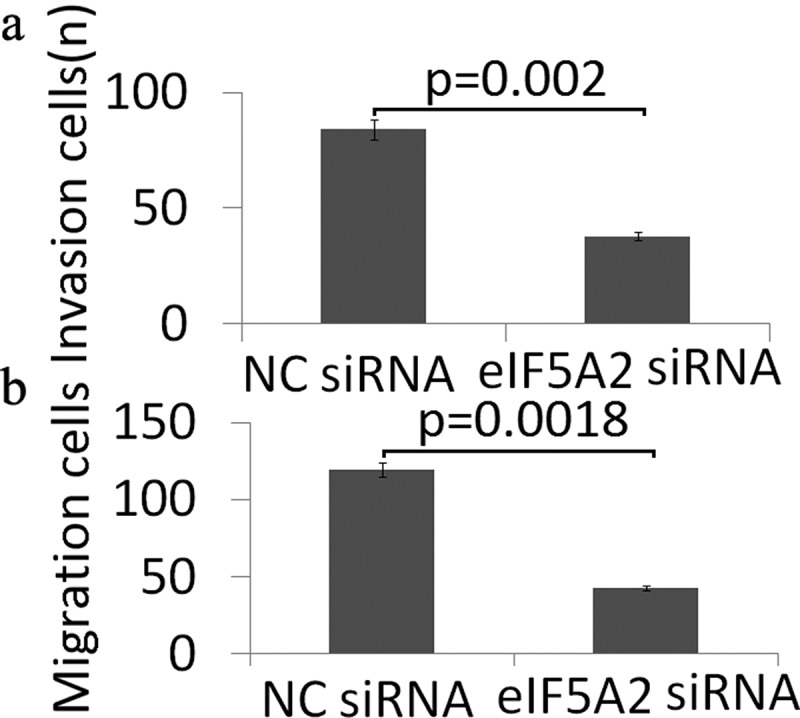
Knockdown of eIF5A2 suppresses BCPAP cell invasion and migration in vitro. BCPAP cells were transiently transfected with either 20 nM NC siRNA or eIF5A2 siRNA for 24 h. The cells were subjected to (a) cell invasion; (b) migration

The affect of targeting eIF5A2 by eIF5A2 siRNA on the migration and the invasive potential of BCPAP cells was evaluated by Transwell assays. As shown in Figure 3(a,b), targeting eIF5A2 significantly inhibited the migration and invasion of BCPAP cells.

### Targeting eIF5A2 inhibits cell growth and lung metastasis

**Figure 4.**
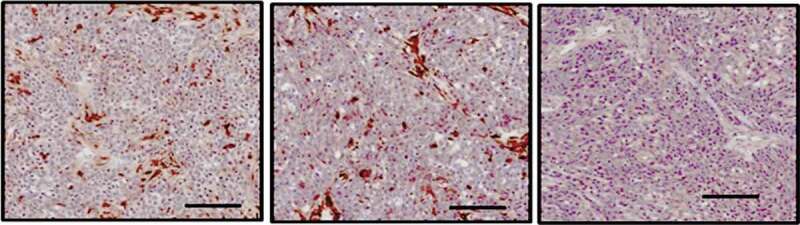
Knockdown of eIF5A2 suppresses BCPAP tumor growth and metastasis in vivo. (a) BCPAP/Lv- shRNA or BCPAP/Lv-eIF5A2 shRNA cells were injected subcutaneously in 4–6 week old BALB/c nude mice. Tumors were measured as indicated. (b) Tissue sections of BCPAP/Lv- shRNA or BCPAP/Lv-eIF5A2 shRNA xenografts were subjected to immunohistochemistry assay for eIF5A2. (c) Tissue sections of BCPAP/Lv- shRNA or BCPAP/Lv-eIF5A2 shRNA xenografts were subjected to TUNEL assay. (d) Tissue sections of BCPAP/Lv- shRNA or BCPAP/Lv-eIF5A2 shRNA xenografts were subjected to immunohistochemistry assay for Ki-67. (e) The metastatic nodes were numbered in the lung

The stably transfected BCPAP/eIF5A2 shRNA cells or BCPAP/NC shRNA cells were inoculated into nude mice subcutaneously for 5 weeks. All of the mice developed xenograft tumors at the subcutaneous injection sites. The BCPAP/eIF5A2 shRNA groups had less tumor mass compared to the BCPAP/NC shRNA groups (Figure 4(a)). Furthermore, eIF5A2 shRNA groups have reduced eIF5A2 (Figure 4(b)) and ki-67 (Figure 4(c)) expression and higher apoptotic cells (Figure 4(d)) compared with the control BCPAP/NC shRNA groups. In addition, there were more metastatic lung nodes in the BCPAP/NC shRNA groups than that in the BCPAP/NC shRNA groups (Figure 4(e)), indicating that targeting eIF5A2 inhibits tumor metastasis *in vivo*.

## Discussion

The expression of eIF5A2 is up-regulated in a variety of malignant tumor tissues, and the up-regulation of eIF5A2 is related to the poor prognosis of the patients with malignant tumors [14,15]. In the study, we first compared the difference of eIF5A2 expression in normal thyroid tissue and PTC tissue as well as the relationship between eIF5A2 expression and PTC clinicopathology. The results showed that eIF5A2 expression was up-regulated in PTC tissues compared to the normal thyroid tissues. and eIF5A2 overexpression was related to extrathyroidal extension, lymph node metastasis, TNM staging, T classification and BRAF V600E mutation. Therefore, overexpression of eIF5A2 may contribute to the progress of PTC. This study is the first to characterize the overexpression of eIF5A2 in PTC tissues.

Previous study has shown that targeting eIF5A2 suppressed cell proliferation in pancreatic cancer cells *in vitro* and *in vivo* [18]. Enhanced eIF5A2, but not eIF5A1, efficiently triggered the growth of hepatocellular carcinoma [19]. We next studied the effect of eIF5A2 on the biological behavior of PTC cells *in vivo* and *in vitro*. Taking BRAF V600E mutant BCPAP cells as an example, the study found that targeting eIF5A2 inhibits the proliferation of BCPAP cells *in vitro* and promotes BCPAP cell apoptosis. At the same time, the invasion and metastasis ability of BCPAP cells is significantly inhibited with eIF5A2 knockout. Further studies demonstrated that targeting eIF5A2 has a significant inhibitory effect on the growth and metastasis of BCPAP cells *in vivo*. The above research data indicated that eIF5A2 is related to the progress of PTC, and eIF5A2 may be the gene target for PTC therapy.

Although eIF5A2 was upregulated in PTC tissues, the mechanism of up-regulation of eIF5A2 expression in PTC is still unclear. The BRAF V600E mutation occurred about 45–50% in PTC, 25–30% in ATC, and none in FTC and benign thyroid neoplasm [20]. BRAF V600E mutation results in a valine-to-glutamic acid change in the BRAF protein, by which to activate BRAF protein kinase and the MAP kinase pathway [21]. In the study, BRAF V600E mutation was enhanced in eIF5A2 overexpressing PTC tissues. And eIF5A2 overexpression was related with tumor invasion. Whether eIF5A2 is regulated by BRAF, or vice versa need further investigation.

PEAK1 is a non-receptor tyrosine kinase that regulates the activity of Src kinase and plays an important role in the occurrence and development of malignant tumors [22–25]. In pancreatic cancer epithelial cells, eIF5A is the upstream regulatory gene of PEAK1, and eIF5A may play a role by regulating PEAK1 signal [18]. In addition, knockdown of eIF5A significantly inhibited endogenous PEAK1 and YAP1 expression, leading to tumor initiation in pancreatic cancer [26]. In our research, whether PEAK1 is regulated by eIF5A2 in PTC cells needs further investigation.

## Conclusion

Our results demonstrated that eIF5A2 was overexpressed in PTC tissues and eIF5A2 overexpression was associated with metastasis and poor prognosis in patients with PTC. We found eIF5A inhibition reduced the PTC cell proliferation and invasive ability *in vivo* and *in vitro*. eIF5A2 provided a novel prognostic marker and therapeutic strategy for PTC patients.

## Highlights

1. eIF5A2 was rich expression in PTC tissues.
2. Enhanced eIF5A2 expression was related with metastasis in patients with PTC.
3. Targeting eIF5A2 inhibited invasiveness and migration *in vitro* and metastasis *in vivo* in PTC cells.
4. Targeting eIF5A2 inhibited cell viability *in vitro* and tumor growth *in vivo* in PTC cells.
